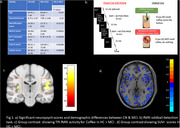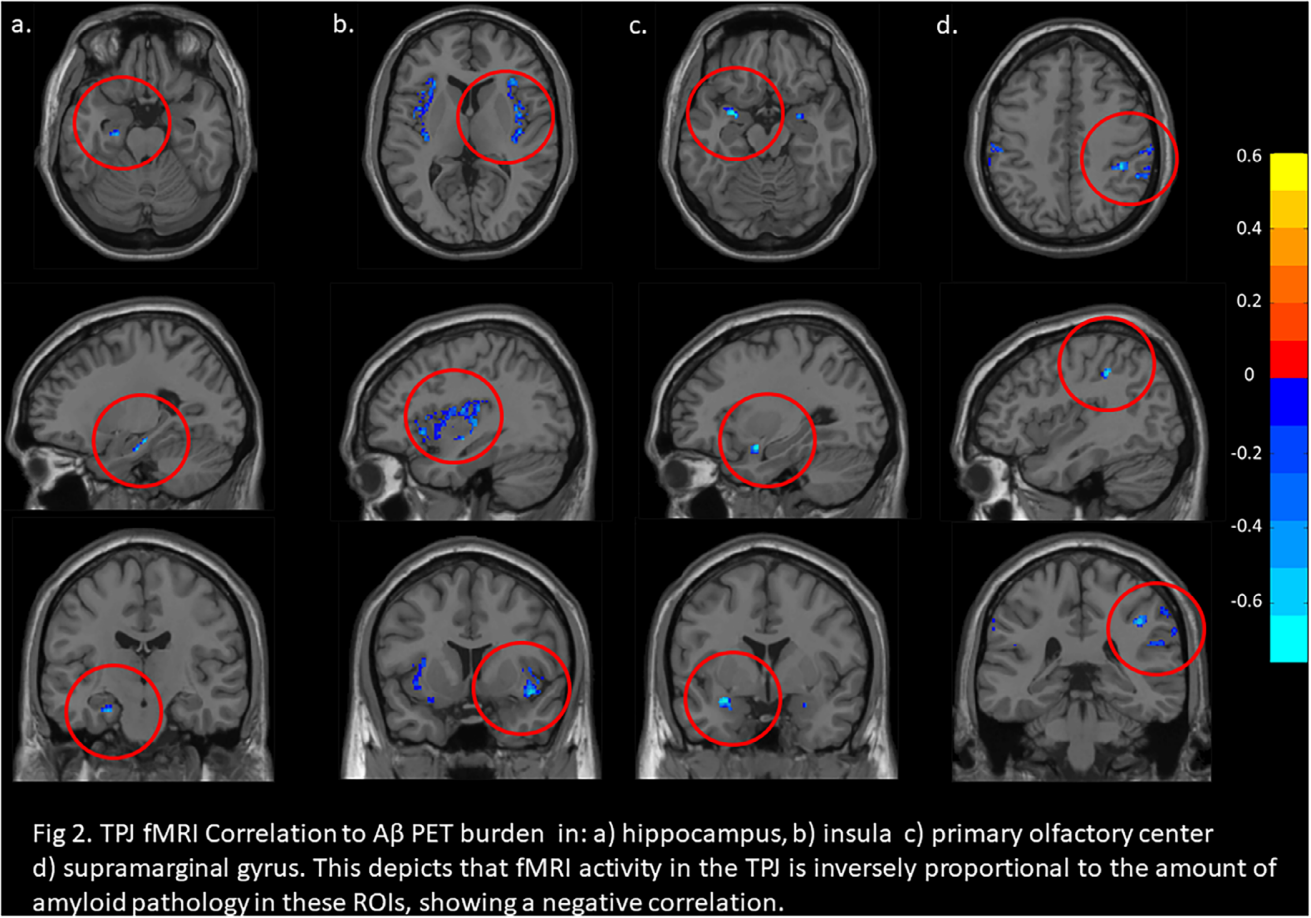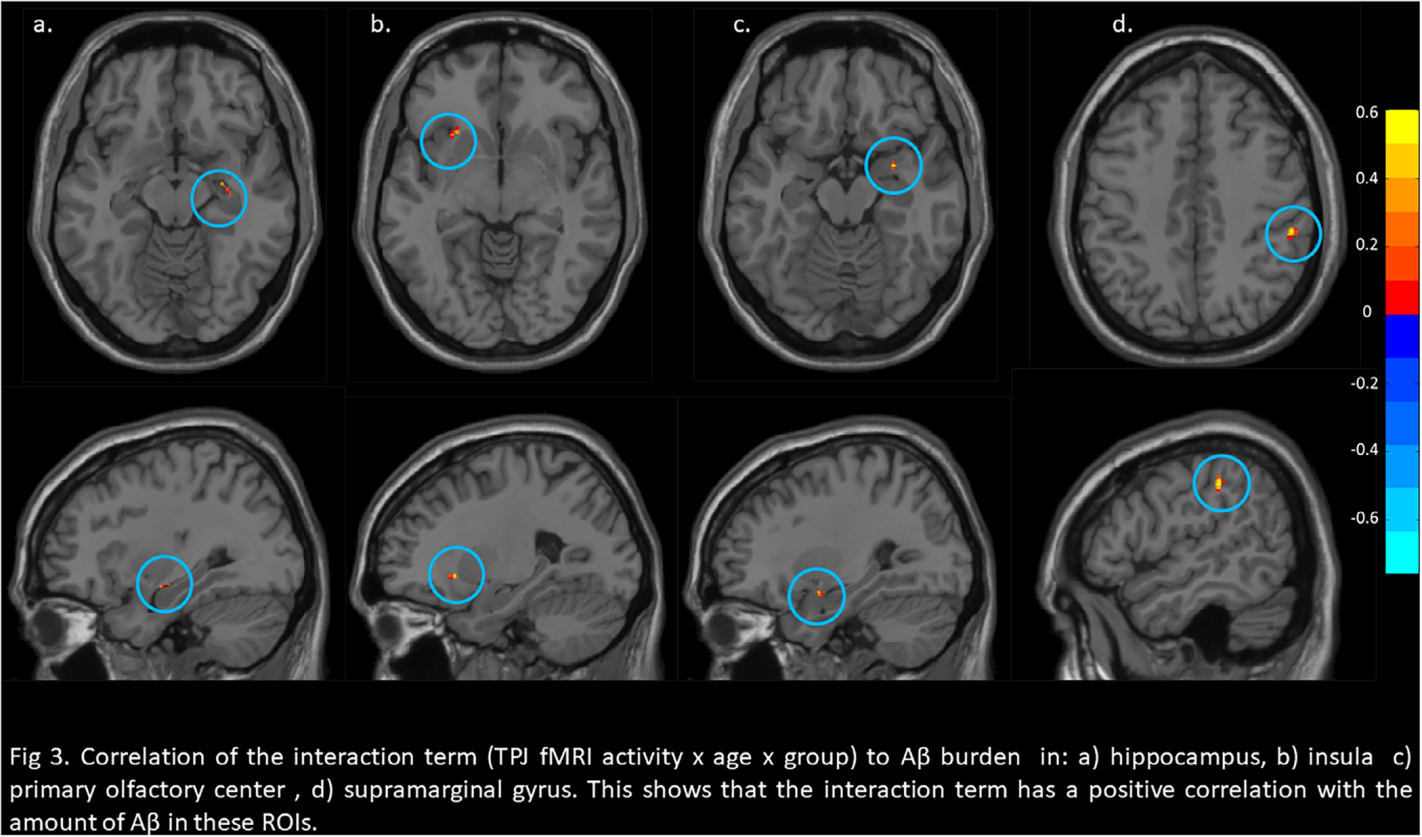# The Role of the Temporoparietal Junction in Alzheimer's Disease

**DOI:** 10.1002/alz70856_103877

**Published:** 2025-12-26

**Authors:** Anupa Manjitha Ekanayake Mudiyanselage, Qing Yang, Senal Peiris, Rommy Elyan, Katie Geesey, Sangam Kanekar, Jens Will, Paul Eslinger, Prasanna Karunanayaka

**Affiliations:** ^1^ Pennsylvania State University College of Medicine, Hershey, PA, USA

## Abstract

**Background:**

In this study, functional magnetic resonance imaging (fMRI) was combined with an olfactory oddball detection task to investigate the role of the temporoparietal junction (TPJ) in odor identification. We expected fMRI activity in TPJ to differ between subjects with mild cognitive impairment (MCI) and healthy controls (HC). We hypothesized that the fMRI activity in the TPJ would correlate negatively with amyloid‐beta (Aβ) standardized uptake value ratio (SUVr) values. We also investigated the interaction term (TPJ fMRI activity *x* age *x* group) on SUVr values in select olfactory regions.

**Method:**

Healthy controls and MCI subjects underwent neuropsychological test evaluations and completed an fMRI oddball odor detection task. The fMRI task required subjects to press a button with their right index finger to identify an oddball (coffee) from a list of distractors (rose, lemon, & bubblegum). Subjects also underwent Aβ‐PET imaging to quantify beta‐amyloid burden in their brains (Figure 1.).

**Result:**

HC and MCI subjects differed in several cognitive and olfactory measures. For the oddball odor, TPJ activity differed between HC and MCI. There were no group differences in TPJ activity between Oddball and non‐oddball odors. The correlation analyses in the combined group showed significant negative correlations between TPJ activity and SUVR values in several olfactory ROIs (Figure 2). The interaction term displayed a positive correlation with SUVr values in the hippocampus, insula, primary olfactory center, and supramarginal gyrus (Figure 3).

**Conclusion:**

TPJ connectivity with other olfactory ROIs decreases with increasing amyloid pathology, which could result in olfactory dysfunction. The correlation between the interaction term and amyloid deposition was positive, but the significance was not strong may be due to the smaller sample size. Taken together, fMRI activity in the TPJ during olfactory fMRI is correlated with SUVr values in the brain, showing a brain‐behavior relationship in MCI.